# Circulatory proteins relate cardiovascular disease to cognitive performance: A mendelian randomisation study

**DOI:** 10.3389/fgene.2023.1124431

**Published:** 2023-02-17

**Authors:** Jian Huang, Dipender Gill, Verena Zuber, Paul M. Matthews, Paul Elliott, Ioanna Tzoulaki, Abbas Dehghan

**Affiliations:** ^1^ Singapore Institute for Clinical Sciences (SICS), Agency for Science, Technology and Research (A*STAR), Singapore, Singapore; ^2^ Department of Epidemiology and Biostatistics, School of Public Health, Imperial College London, London, United Kingdom; ^3^ Department of Brain Sciences, Faculty of Medicine, Imperial College London, London, United Kingdom; ^4^ UK Dementia Research Institute at Imperial College London, London, United Kingdom; ^5^ MRC Centre for Environment and Health, School of Public Health, Imperial College London, London, United Kingdom; ^6^ Department of Hygiene and Epidemiology, University of Ioannina Medical School, Ioannina, Greece

**Keywords:** proteins, cardiovascular disease, cognition, mendelian randomisation, myeloperoxidase (MPO)

## Abstract

**Background and objectives:** Mechanistic research suggests synergistic effects of cardiovascular disease (CVD) and dementia pathologies on cognitive decline. Interventions targeting proteins relevant to shared mechanisms underlying CVD and dementia could also be used for the prevention of cognitive impairment.

**Methods:** We applied Mendelian randomisation (MR) and colocalization analysis to investigate the causal relationships of 90 CVD-related proteins measured by the Olink CVD I panel with cognitive traits. Genetic instruments for circulatory protein concentrations were obtained using a meta-analysis of genome-wide association studies (GWAS) from the SCALLOP consortium (N = 17,747) based on three sets of criteria: 1) protein quantitative trait loci (pQTL); 2) *cis*-pQTL (pQTL within ±500 kb from the coding gene); and 3) brain-specific *cis*-expression QTL (*cis*-eQTL) which accounts for coding gene expression based on GTEx8. Genetic associations of cognitive performance were obtained from GWAS for either: 1) general cognitive function constructed using Principal Component Analysis (N = 300,486); or, 2) *g* Factor constructed using genomic structural equation modelling (N = 11,263–331,679). Findings for candidate causal proteins were replicated using a separate protein GWAS in Icelanders (N = 35,559).

**Results:** A higher concentration of genetically predicted circulatory myeloperoxidase (MPO) was nominally associated with better cognitive performance (*p* < 0.05) using different selection criteria for genetic instruments. Particularly, brain-specific *cis*-eQTL predicted MPO, which accounts for protein-coding gene expression in brain tissues, was associated with general cognitive function (β_Wald_ = 0.22, P_Wald_ = 2.4 × 10^−4^). The posterior probability for colocalization (PP.H4) of MPO pQTL with the *g* Factor was 0.577. Findings for MPO were replicated using the Icelandic GWAS. Although we did not find evidence for colocalization, we found that higher genetically predicted concentrations of cathepsin D and CD40 were associated with better cognitive performance and a higher genetically predicted concentration of CSF-1 was associated with poorer cognitive performance.

**Conclusion:** We conclude that these proteins are involved in shared pathways between CVD and those for cognitive reserve or affecting cognitive decline, suggesting therapeutic targets able to reduce genetic risks conferred by cardiovascular disease.

## Introduction

The main protein biomarkers identified for dementia include amyloid precursor protein and tau protein (O'Brien and Wong, 2011). Clinical trials of anti-amyloid and anti-tau intervention for AD have been conducted but meaningfully effective drugs are still not available (Congdon and Sigurdsson, 2018; Huang et al., 2020), and their causal role is under question (Kametani and Hasegawa, 2018; Morris et al., 2018; Thomas et al., 2020; Sturchio et al., 2021). Accumulating evidence has suggested a link between cardiovascular disease (CVD) and dementia (Paciaroni and Bogousslavsky, 2013). Classical cardiovascular risk factors, such as smoking, diabetes, hypertension, obesity, and physical inactivity contribute to a higher risk of cognitive impairment and dementia (Juul Rasmussen et al., 2020; Yaffe et al., 2020). Mechanistic research suggests that the impact of CVD risk burden on dementia pathologies could lead to cognitive decline (Attems and Jellinger, 2014; Santos et al., 2017). Plasma proteins for CVD related to several biological pathways that may be responsible for this have been identified. Confident identification of causal relationships between these protein biomarkers and cognition would support the development of pathway-specific treatment (Ho et al., 2018; Wallentin et al., 2021). With strong evidence that such proteins play shared causal roles in the mechanisms underlying CVD and cognition, interventions targeting these proteins and their pathways could be developed to prevent, slow or reverse disease progression and cognitive impairment.

Some recent trials have shifted the focus to the early stages of AD or mild cognitive impairment (Congdon and Sigurdsson, 2018). Given the high failure rate of the translation of preclinical drug candidates in animal models into treatment in humans, a more cost-effective strategy for drug discovery for cognitive impairment is needed. The recent development of genotyping and proteomic technologies has enabled the detection of protein quantitative trait loci (pQTL) in large-scale genome-wide association studies (GWAS) (Folkersen et al., 2020). Albeit most GWAS in human subjects interrogate plasma pQTL (rather than brain or brain cell-specific pQTL), they still can be used as genetic instruments for instrumental variable analyses such as Mendelian randomisation (MR) (Gill et al., 2021). MR analysis uses genetic variants, which are randomised at conception, to mimic random allocation in clinical trials and investigate the causal relationship between a risk factor and an outcome (Harris et al., 2020). It is less susceptible to confounding or reverse causation than conventional observational studies and can be used to identify potential molecular targets for intervention (Harris et al., 2020). In this study, we applied MR analyses to assess the associations of a wide range of plasma proteins, which are known to associate with CVD, with cognitive traits.

## Methods

### Genetic associations of concentrations of plasma proteins

Genetic associations of 90 proteins in plasma were obtained from a GWAS meta-analysis comprised of 13 cohorts of European ancestry (SCALLOP consortium; average per-protein sample size, 17,747) (Folkersen et al., 2020). Relative protein quantification was measured using the Olink proximity extension assay (PEA) cardiovascular (CVD) I panel (Assarsson et al., 2014). The selected protein biomarkers are associated with cardiovascular risk or prognosis in human observational studies and animal models (Assarsson et al., 2014; Folkersen et al., 2020). The Olink PEA CVD I panel returns normalized protein expression (NPX) values (on the log2 scale so that each one-unit difference in NPX indicates a doubling of protein concentration). NPX values were rank-based inverse-normalised to unit variance for the genetic association analysis. Supplementary Table S1 listed the proteins under investigation in this study.

To replicate the findings, we obtained the summary statistics for the genetic instruments from a separate GWAS of protein concentrations measured by SomaScan assay among 35,559 Icelanders (Ferkingstad et al., 2021). The SomaScan assay provides an aptamer-based measurement of the relative binding of a putative target protein to each aptamer in relative fluorescence units (Ferkingstad et al., 2021). Because of the methodological differences, the effect sizes from analyses based on Olink and Somalogic data cannot be compared directly.

### Genetic associations of cognition

We obtained the effect estimates for the association of genetic instruments with two cognitive traits constructed using different plasma protein datasets (Davies et al., 2018; de la Fuente et al., 2021). A GWAS meta-analysis for general cognitive function (N = 300,486, age 16–102 years) was performed based on the first unrotated principal component of multiple cognitive tasks from 57 population-based cohorts of the Cohorts for Heart and Aging Research in Genomic Epidemiology (CHARGE) and the Cognitive Genomics Consortium (COGENT) consortia and the verbal and numerical reasoning test in UK Biobank (Davies et al., 2018). A GWAS for the general factor of intelligence (*g* Factor) was performed based on seven different cognitive tests (N = 11,263–331,679, age 40–70  years at first assessment) in UK Biobank (de la Fuente et al., 2021). For this association analysis, a univariate GWAS was performed for each of the seven standardised cognitive test scores and the univariate summary statistics then were used to conduct a multivariate GWAS using genomic structural equation modelling (Genomic SEM) (Grotzinger et al., 2019). In Genomic SEM, the target trait represents the genetic components of the individual GWAS traits (de la Fuente et al., 2021). A higher *g* Factor score indicates better performance in cognitive tasks. Supplementary Table S2 shows the details of GWAS of cognition.

### Bidirectional two-sample univariable mendelian randomisation

We performed a two-sample univariable MR to investigate the associations of each protein with either general cognitive function or the *g* Factor. For each MR analysis, we selected genetic instruments for each protein using three sets of criteria. Under the first set of criteria, we selected any single-nucleotide polymorphisms (SNPs) throughout the genome associated with the protein of interest at a *p*-value<5 × 10^−8^ (both *cis*- and *trans*-). For the proteins with less than three SNPs under these criteria, we loosened the *p*-value threshold to 5 × 10^−6^. We referred to this set of instruments as pQTL instrumental variables. Under the second set of criteria, we selected *cis*-pQTL (i.e., pQTL within ±500 kb from the protein coding gene) associated with the protein of interest at *p*-value<5 × 10^−6^. To test directly for potentially causal associations with brain protein expression, we, under the third set of criteria, leveraged the information from GTEx8 expression quantitative trait loci (eQTL) by meta-analysing eQTL from 13 brain tissues (Supplementary Table S3; sample sizes ranged from 139 to 255). We selected SNPs located within ±500 kb from the coding gene, associated with expression of the coding gene at *p*-value<10^−4^, and associated with circulating protein concentration at *p*-value<0.05. We defined these as brain-specific *cis*-eQTL. For all three sets of criteria, we only included SNPs with a minor allele frequency (MAF) greater than 5% and F-statistics greater than 10. Correlated SNPs (*r* ^2^ ≥ 0.001) were excluded by keeping the one with the smallest *p*-value.

We also performed a two-sample MR to investigate whether cognitive traits or their genetic liability affect the plasma concentrations of selected marker proteins. We selected independent SNPs (*r* ^2^ < 0.001) associated with the cognitive traits of interest at *p*-value<5 × 10^−8^, MAF greater than 5%, and F-statistics>10 as genetic instruments.

For each MR analysis, genetic associations of the selected genetic instruments with the outcome of interest were obtained from the corresponding GWAS. We estimated the SNP-specific effects using the Wald ratio (Burgess et al., 2017) and pooled the SNP-specific estimates using inverse-variance weighted (IVW) for the MR effect estimates (Lawlor et al., 2008). We used IVW fixed-effects model for the analyses with two or three SNPs and IVW random-effects model for the analyses with more than three SNPs (Bowden et al., 2017). We also performed two sensitivity methods (weighted median (WM) and MR-Egger regression) to assess the robustness of MR estimates and horizontal pleiotropic effects for analyses with at least three SNPs (Bowden et al., 2016; Burgess and Thompson, 2017). Potential outlier SNPs were identified using MR-PRESSO and excluded from the analysis (Verbanck et al., 2018). We accounted for multiple comparisons using Bonferroni correction for 90 plasma proteins and two directions with a *p*-value threshold of 0.05/180 = 0.0003. We prioritised candidate proteins by accounting for the findings based on all three sets of criteria (i.e., pQTL, *cis*-pQTL, and brain-specific *cis*-eQTL)

### Colocalization

We performed colocalization to estimate the posterior probability of the hypothesis that a cognitive trait shares the same causal variant with a candidate protein (i.e., PP.H4) using both pQTL and brain-specific eQTL (Zuber et al., 2022). Specifically, we focused on the genomic region within ±50 kb from the protein coding gene of interest. Given that we performed colocalization for the candidate MR findings, here we tested only whether the common causal variant is more likely than other hypotheses (see Supplementary Table S8). Evidence of colocalization provides complementary information on causal relationships since distinct causal variants for the exposure and outcome of interest could lead to a violation of the exchangeability assumption in MR analysis (Zuber et al., 2022). We considered a PP.H4>0.5 as evidence for colocalization (Giambartolomei et al., 2014).

### Replication analysis

To validate our findings, we repeated the analysis of findings in our main MR analyses using summary statistics from a GWAS of putative protein concentrations measured using the SomaScan assay (Ferkingstad et al., 2021). Specifically, we conducted MR and colocalisation analysis for macrophage colony-stimulating factor 1 (CSF-1), cathepsin D (CTSD), and myeloperoxidase (MPO). We were not able to extend the analysis to tumour necrosis factor receptor superfamily member 5 (TNFRSF5, commonly known as CD40) as this protein was not measured in the Icelandic GWAS. We also performed additional analyses for IL-34 and CSF-1 receptor (CSF1R), since IL-34 and CSF-1 are both ligands of CSF-1 receptor.

### Statistical software

All analyses were performed in R 3.6.1. Bidirectional MR analyses were performed using the *TwoSampleMR* and *MR-PRESSO* packages (Verbanck et al., 2018). Colocalization was performed using the *coloc* package (Giambartolomei et al., 2014).

## Results

**FIGURE 1:**
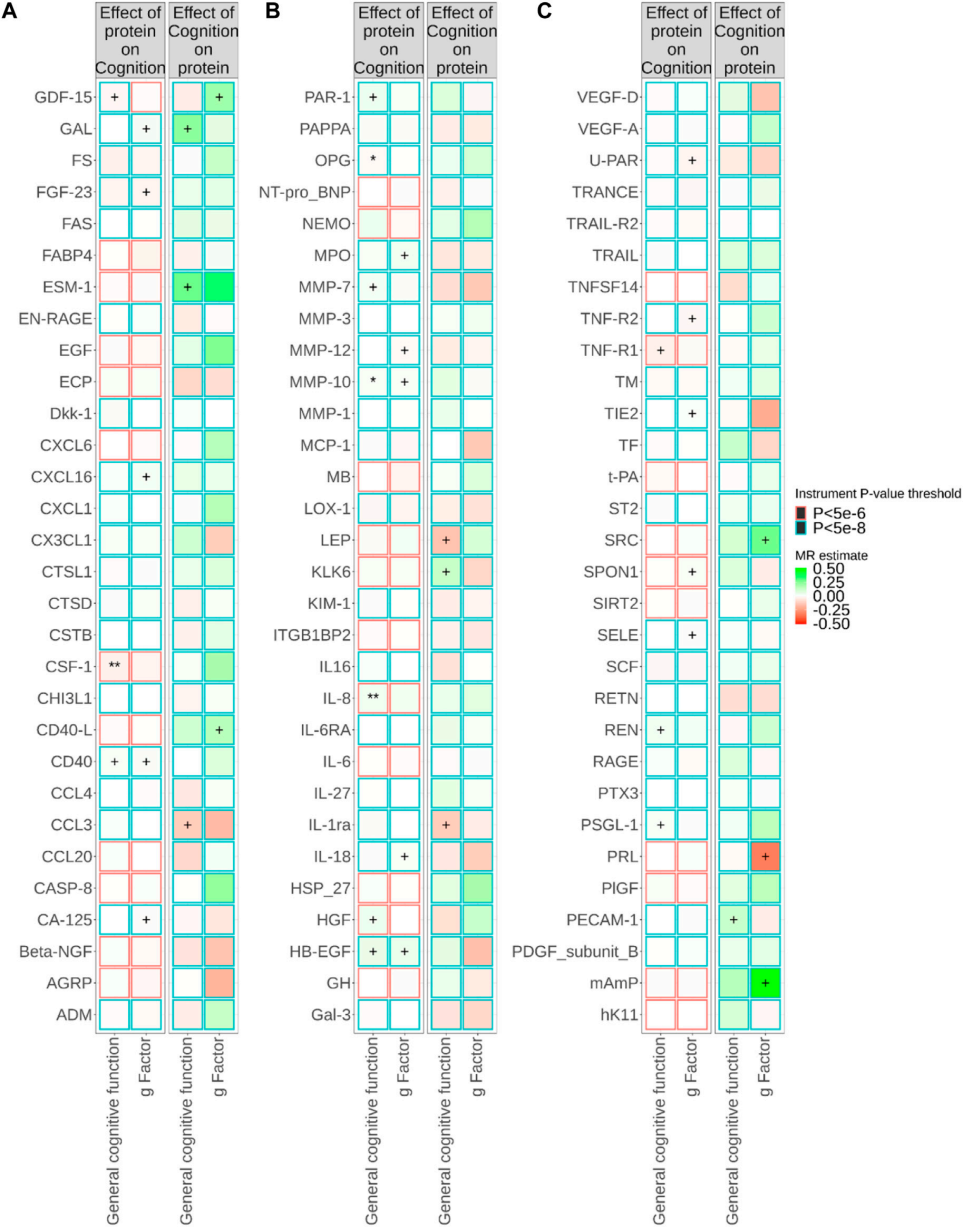
Relationships between plasma protein concentrations and cognitive traits from bidirectional Mendelian randomisation (MR). MR estimate was obtained from inverse-variance weighted (IVW) method. The symbol + indicates an association with a *p*-value smaller than 0.05, the symbol * indicates an association with a *p*-value smaller than 0.05/90, and the symbol ** indicates an association with a *p*-value smaller than 0.05/180. Due to the large number of proteins, MR results for different proteins are presented in **(A–C)** in this figure.

**FIGURE 2:**
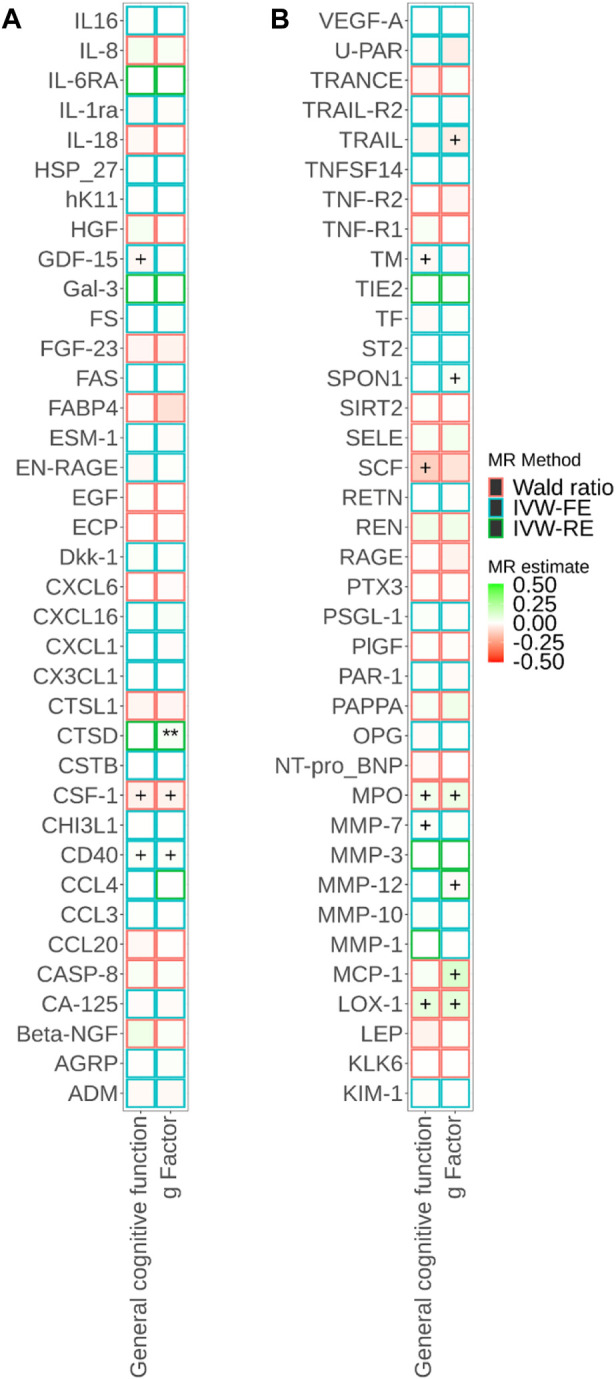
Associations of cis-pQTL-predicted plasma protein concentrations with cognitive traits from Mendelian randomisation (MR). MR estimate was obtained from the Wald ratio method (N_SNP_ = 1) and inverse-variance weighted (IVW) method (N_SNP_>1). The symbol + indicates an association with a *p*-value smaller than 0.05, the symbol * indicates an association with a *p*-value smaller than 0.05/90, and the symbol ** indicates an association with a *p*-value smaller than 0.05/180. Due to the large number of proteins, MR results for different proteins are presented in Panels a and b in this figure.

**FIGURE 3:**
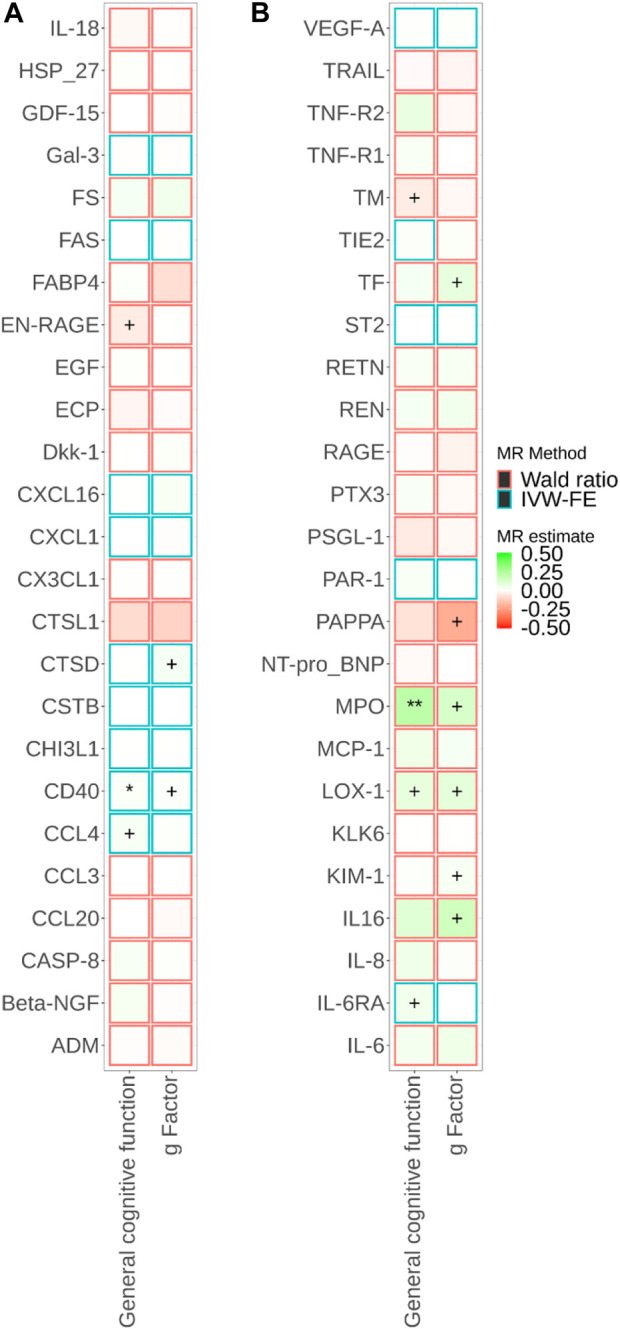
Associations of brain-specific *cis*-eQTL-predicted plasma protein concentrations with cognitive traits from Mendelian randomisation (MR). MR estimate was obtained from the Wald ratio method (N_SNP_ = 1) and inverse-variance weighted (IVW) method (N_SNP_>1). The symbol + indicates an association with a *p*-value smaller than 0.05, the symbol * indicates an association with a *p*-value smaller than 0.05/90, and the symbol ** indicates an association with a *p*-value smaller than 0.05/180. Due to the large number of proteins, MR results for different proteins are presented in Panels a and b in this figure.

Figure 1; Supplementary Tables S4, S5 show the relationships between plasma proteins and cognitive traits defined by the bidirectional univariable MR analyses. Among the 180 univariable MR analyses for the associations of pQTL-predicted plasma protein concentrations with cognitive traits, 115 had at least three SNPs with *p*-value<5 × 10^−8^ and *r* ^2^ < 0.001 (F-statistics ranged from 30 to 7,577). For the remaining 65 univariable MR analyses (33 proteins), we selected SNPs with *p*-value<5 × 10^−6^ and *r* ^2^ < 0.001 (F-statistics ranged from 21 to 1,811). We successfully identified *cis-*pQTL instruments for 75 circulating proteins and brain-specific *cis*-eQTL instruments for 50 circulating proteins (Figures 2, 3; Supplementary Tables S6, S7 **)**. Table 1 shows that genetically MPO, CSF-1, CTSD, and CD40 were associated with cognitive performance under different instrument selection criteria.

**TABLE 1:** Associations of circulating proteins with cognitive performance based on Mendelian randomisation analyses.

Exposure	Instrument type	Number of SNPs	Method	Beta	SE	*p*-value^a^	95% CI (lower)	95% CI (upper)	Heterogeneity *p*-value	Pleiotropy *p*-value
**Protein → General cognitive function**										
CD40	pQTL	3	IVW-FE	0.019	0.006	0.001*	0.007	0.030	0.696	-
			WM	0.019	0.006	0.001*	0.008	0.030	-	-
			MR-Egger	0.024	0.008	0.216	0.007	0.040	0.923	0.553
	cis-pQTL	3	IVW-FE	0.017	0.006	0.003*	0.006	0.029	0.021	-
			WM	0.019	0.006	0.001*	0.008	0.030	-	-
			MR-Egger	0.031	0.011	0.222	0.009	0.053	0.166	0.332
	cis-eQTL	2	IVW-FE	0.021	0.006	4.4E-04*	0.009	0.032	-	-
CSF-1	pQTL	8	IVW-RE	−0.033	0.008	4.5E-05**	−0.048	−0.017	0.814	-
			WM	−0.036	0.013	0.004*	−0.061	−0.011	-	-
			MR-Egger	−0.042	0.025	0.141	−0.091	0.007	0.741	0.690
	cis-pQTL	1	Wald ratio	−0.037	0.015	0.013*	−0.065	−0.008	-	-
CTSD	pQTL	7	IVW-RE	−0.008	0.010	0.409	−0.028	0.011	0.081	-
			WM	0.001	0.009	0.935	−0.017	0.019	-	-
			MR-Egger	0.010	0.016	0.579	−0.022	0.042	0.143	0.234
	cis-pQTL	4	IVW-RE	0.001	0.007	0.828	−0.011	0.014	0.614	-
			WM	0.004	0.009	0.622	−0.013	0.022	-	-
			MR-Egger	0.008	0.015	0.650	−0.022	0.038	0.465	0.653
	cis-eQTL	3	IVW-FE	0.005	0.010	0.608	−0.014	0.024	0.945	-
			WM	0.005	0.010	0.603	−0.014	0.024	-	-
			MR-Egger	0.003	0.014	0.852	−0.024	0.030	0.774	0.888
MPO	pQTL	10	IVW-RE	0.020	0.014	0.157	−0.008	0.047	0.051	-
			WM	0.038	0.014	0.006*	0.011	0.065	-	-
			MR-Egger	0.052	0.028	0.103	−0.003	0.107	0.082	0.233
	cis-pQTL	1	Wald ratio	0.045	0.018	0.010*	0.011	0.080	-	-
	cis-eQTL	1	Wald ratio	0.219	0.059	2.0E-04**	0.104	0.335	-	-
**Protein → g Factor**										
CD40	pQTL	3	IVW-FE	0.016	0.006	0.012*	0.004	0.028	0.332	-
			WM	0.016	0.006	0.014*	0.003	0.028	-	-
			MR-Egger	0.015	0.014	0.470	−0.012	0.042	0.139	0.945
	cis-pQTL	3	IVW-FE	0.017	0.006	0.009*	0.004	0.029	0.469	-
			WM	0.016	0.006	0.011*	0.004	0.029	-	-
			MR-Egger	0.014	0.010	0.399	−0.006	0.035	0.241	0.804
	cis-eQTL	2	IVW-FE	0.016	0.007	0.012*	0.004	0.029	-	-
CSF-1	pQTL	10	IVW-RE	−0.023	0.013	0.082	−0.049	0.003	0.215	-
			WM	−0.035	0.015	0.017*	−0.064	−0.006	-	-
			MR-Egger	−0.048	0.031	0.161	−0.109	0.013	0.208	0.398
	cis-pQTL	1	Wald ratio	−0.035	0.016	0.032*	−0.067	−0.003	-	-
CTSD	pQTL	6	IVW-RE	0.018	0.012	0.145	−0.006	0.042	0.104	-
			WM	0.028	0.010	0.008*	0.007	0.048	-	-
			MR-Egger	0.041	0.016	0.068	0.009	0.073	0.279	0.149
	cis-pQTL	4	IVW-RE	0.026	0.005	8.4E-08**	0.017	0.036	0.853	-
			WM	0.028	0.010	0.005*	0.008	0.048	-	-
			MR-Egger	0.030	0.017	0.213	−0.003	0.063	0.707	0.790
	cis-eQTL	3	IVW-FE	0.032	0.011	0.004*	0.010	0.053	0.189	-
			WM	0.031	0.011	0.004*	0.010	0.053	-	-
			MR-Egger	0.021	0.025	0.546	−0.027	0.069	0.118	0.655
MPO	pQTL	9	IVW-RE	0.031	0.014	0.026*	0.004	0.059	0.139	-
			WM	0.052	0.016	0.001*	0.020	0.084	-	-
			MR-Egger	0.079	0.023	0.011*	0.034	0.124	0.485	0.047
	cis-pQTL	1	Wald ratio	0.064	0.019	0.001*	0.028	0.101	-	-
	cis-eQTL	1	Wald ratio	0.138	0.064	0.030*	0.013	0.264	-	-

^a^ “**” indicates a *p*-value smaller than 0.05/180, and “*” indicates a *p*-value smaller than 0.05.

Abbreviations: tumour necrosis factor receptor superfamily member 5 (TNFRSF5, commonly known as CD40); confidence interval (CI); macrophage colony-stimulating factor 1 (CSF-1); cathepsin D (CTSD); expression quantitative trait loci (eQTL); inverse-variance weighted fixed-effect (IVW-FE); inverse-variance weighted random-effect (IVW-RE); myeloperoxidase (MPO); protein quantitative trait loci (pQTL); standard error (SE); single-nucleotide polymorphisms (SNPs); weighted median (WM).

Supplementary Figures S1, S2 show that nominal significant associations for pQTL predicted plasma protein concentration with cognitive traits (*p*-value for IVW <0.05) were consistent in the direction using sensitivity methods (WM and MR-Egger). After accounting for multiple comparisons (P_IVW_<0.05/180), a higher concentration of pQTL-predicted CSF-1 was associated with poorer general cognitive function (β_IVW_ = −0.03; 95% confidence interval (CI) −0.05, −0.02; P_IVW_ = 4.5 × 10^−5^). A higher concentration of *cis*-pQTL-predicted CSF-1 was nominally associated with both poorer general cognitive function (β_Wald_ = −0.04; 95% CI -0.065, −0.008; P_Wald_ = 0.01) and lower *g* Factor score (β_Wald_ = −0.04; 95% CI -0.067, −0.003; P_Wald_ = 0.03). The only two brain-specific *cis*-eQTL for CSF-1 were both rare variants (MAF = 2%), thus MR analysis using brain-specific *cis*-eQTL was not performed. However, there was weak evidence for colocalization for CSF-1 with cognitive traits (Supplementary Table S8; PP.H4<0.1 for both pQTL and eQTL).

A higher concentration of pQTL-predicted IL-8 was associated with better general cognitive function (β_IVW_ = 0.03; 95% CI 0.02, 0.05; P_IVW_ = 2.4 × 10^−5^). *Cis*-pQTL and brain-specific *cis*-eQTL instruments were not identified for IL-8. We did not find supporting evidence for colocalization for IL-8 with cognitive traits (Supplementary Table S8; PP.H4<0.1 for both pQTL and eQTL).

Although pQTL-predicted CTSD plasma concentration was not associated with cognitive traits, a higher concentration of *cis*-pQTL-predicted CTSD was associated with a higher *g* Factor score after accounting for multiple comparisons (β_IVW_ = 0.03; 95% CI 0.02, 0.04; P_IVW_ = 8.4 × 10^−8^), and a higher concentration of brain-specific *cis*-eQTL-predicted CTSD was nominally associated with higher *g* Factor score (β_IVW_ = 0.03; 95% CI 0.01, 0.05; P_IVW_ = 0.004). MR estimates using sensitivity methods showed a consistent direction and similar effect size for these associations and MR-Egger did not suggest horizontal pleiotropy. However, colocalization for CTSD with *g* Factor was not found (Supplementary Table S8; PP.H4<0.1 for both pQTL and eQTL).

**FIGURE 4:**
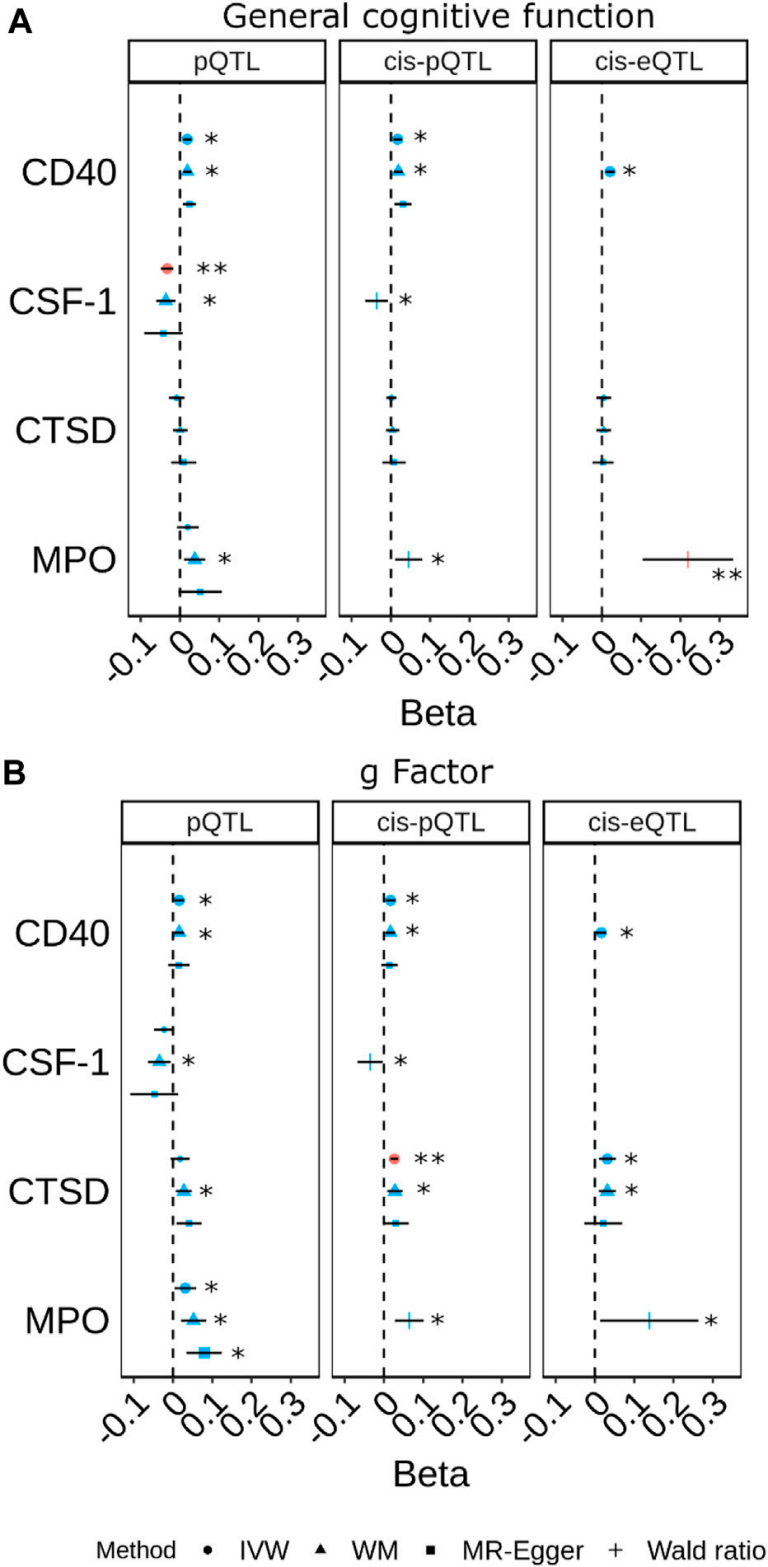
Forest plots for the most prominent findings in univariable Mendelian randomisation (MR) for the associations of genetically predicted plasma protein concentrations with cognitive traits (**(A)** general cognitive function. **(B)** g Factor). Each panel indicates the estimates from MR analysis based on three sets of selection criteria for genetic instruments. The shape in red and the double asterisk symbol (**) indicate an association with a *p*-value<0.05/180. The symbol in blue and larger in size and the asterisk symbol (*) indicate an association with a *p*-value<0.05. (Inverse-variance weighted (IVW); weighted median (WM)).

The pQTL, cis-pQTL, and brain-specific cis-eQTL predicted CD40 and MPO were consistently nominally associated with better cognitive performance in the MR analyses (Figure 4; Supplementary Tables S4–S6). Particularly, brain-specific cis-eQTL-predicted MPO was associated with general cognitive function after accounting for multiple comparisons (β_Wald_ = 0.22; 95% CI 0.10, 0.33; P_Wald_ = 2.4 × 10^−4^). PP.H4 was 0.577 for MPO pQTL with *g* Factor (Supplementary Table S8).

Genetically predicted cognition was nominally associated with concentrations of plasma proteins (Figure 1; Supplementary Table S5). However, we did not find evidence supporting an influence of genetically predicted cognition on plasma concentrations of CSF-1, IL-8, CTSD, CD40, and MPO.

Using genetic associations from the Icelandic GWAS, a higher concentration of cis-pQTL-predicted MPO was nominally associated with better cognitive performance (Supplementary Table S9 **)** measured as the general cognitive function (β_IVW_ = 0.02; 95% CI 0.003, 0.04; P_IVW_ = 0.02) or *g* Factor (β_IVW_ = 0.02; 95% CI 0.002, 0.04; P_IVW_ = 0.03). Consistent with our main analysis based on the SCALLOP GWAS, the PP.H4 was 0.577 for MPO pQTL (Icelandic GWAS) with the *g* Factor. In addition, a higher concentration of pQTL-predicted IL-34 (a ligand of CSF-1 receptor) was nominally associated with better cognitive performance but there was no evidence of colocalization (PP.H4<0.1).

## Discussion

By aggregating evidence from MR (with three sets of instrument selection criteria) and colocalization, our study found that a higher concentration of genetically predicted MPO was associated with better cognitive performance. Genetically predicted CSF-1, CTSD, and CD40 were associated with cognitive performance, but causal relationships were not supported by colocalization. Conversely, we found no evidence suggesting cognitive traits or their genetic liability to affect circulating concentrations of these proteins.

Our associations of circulating proteins with general cognitive function and *g* Factor score were not always consistent. This may be attributed to the different methods utilised to construct the two cognitive traits. While *g* Factor score was constructed based on seven cognitive tests using Genomic SEM, it may have captured greater contributions from reaction time and memory, given the larger sample sizes for these tests (e.g., N = 331,679 for the memory pairs-matching test, N = 330,024 for reaction time, and N = 11,356 for the matrix pattern recognition test) (de la Fuente et al., 2021). General cognitive function was constructed based on a wider range of cognitive tests using principal component analysis, which showed a stronger genetic correlation with educational attainment (Davies et al., 2018; de la Fuente et al., 2021). Nevertheless, we observed consistent associations for genetically predicted MPO with both these two cognitive traits in our MR analyses.

In this study, we observed a direct association between circulatory concentrations of MPO and cognitive function. In agreement with this finding, studies have reported an association of a functional polymorphism in the promoter region of the *MPO* gene (rs2333227, G-463A) with cognitive function and risk of Alzheimer’s disease (Crawford et al., 2001; Combarros et al., 2002; Pope et al., 2006; Talarowska et al., 2015). The *MPO AA* genotype, which decreases the production of myeloperoxidase, was associated with cognitive decline among older adults (aged 70–79 years) (Pope et al., 2006). However, both *MPO* expression and protein levels in middle-aged adults (aged 20–67 years, mean = 40) were found to be associated with worse cognitive function as assessed using Trail Making Test, Stroop Test, Verbal Fluency Test, and Auditory-Verbal Learning Test (Talarowska et al., 2015). Inconsistent findings have been reported for MPO and the risk of Alzheimer’s disease. The *MPO GG* genotype (increasing production of myeloperoxidase) was associated with a higher risk for Alzheimer’s disease among Caucasians but not Hispanics (Crawford et al., 2001). Sex-specific risk for Alzheimer’s disease among individuals with the *MPO GG* genotype also has been reported (Reynolds et al., 1999). In addition, circulating MPO was higher among individuals with mild cognitive impairment and Alzheimer’s patients compared to healthy controls (Folkersen et al., 2020). This may suggest the role of MPO in cognitive performance varies by age and sex. We were not able to perform sex-specific MR analysis given that sex-specific GWAS for Alzheimer’s disease with a large sample size was not available. A higher concentration of circulating MPO was associated with an increased incidence and poorer prognosis of CVD (Ramachandra et al., 2020). Further investigation is needed to elucidate the mechanisms underlying the effects of MPO on cognition and CVD.

We also found a direct association between circulatory concentrations of CD40 and cognitive performance. A published study by Ye et al. (2019) showed that the CD40 concentrations in the cerebrospinal fluid were lower among patients with mild Alzheimer’s disease compared with healthy controls and MCI patients. Increased expression of CD40 is associated with microglial activation, which has been found to contribute to cognitive impairments and the pathogenesis of Alzheimer’s disease (Hamelin et al., 2018; Zhang et al., 2021). Inhibition of the CSF-1 receptor reduces microglial activation (Olmos-Alonso et al., 2016). A previous study reported a higher concentration of plasma CTSD among AD patients with less severe cognitive impairment (Kim et al., 2021). Increased expression of CTSD, as lysosomal protease that degrades both amyloid-beta and tau proteins, is part of an adaptive response to AD-related neurodegenerative pathology (Cataldo et al., 1995; Suire et al., 2020). Our findings of higher genetically predicted CD40 and CTSD associated with better cognitive performance and higher genetically predicted CSF-1 with poorer general cognitive function are consistent with this. CD40, CSF-1, and CTSD also have been reported to play a role in CVD, possibly through their roles in mediating inflammation (Gorelick, 2010; Ozawa et al., 2017; Sjaarda et al., 2018; Daub et al., 2020; Hoes et al., 2020). This supports the shared mechanisms underlying CVD, cognitive impairment, and Alzheimer’s disease. Further research on repurposing CVD drugs for cognitive impairment and Alzheimer’s disease is warranted.

In brief, our findings highlight a number of CVD-related proteins that may play a role in building the cognitive reserve or cognitive decline based on MR analyses with rigorously selected genetic instruments. We improved the validity of genetic instruments by using stringent *p*-value thresholds and performed sensitivity analyses using *cis*-pQTL located near the protein coding gene of interest and brain-specific *cis*-eQTL leveraging gene expression in brain tissues. Sensitivity MR methods showed consistent results and bidirectional MR did not suggest reverse causation. However, we recognise the limitations of our analyses. First, our analysis was based on the GWAS of circulatory protein concentrations measured using Olink CVD I panel. Large GWAS of other CVD-related proteins measured using CVD II or III panel was not available at the time of analysis. Second, the effect sizes from analyses based on the Olink protein GWAS and the Icelandic GWAS (SomaScan assay) cannot be compared directly. However, MPO measured by the two technologies are expected to be highly correlated (Pietzner et al., 2021). Third, colocalization provides moderate evidence for a common causal variant between MPO or CD40 and cognitive traits (PP.H4: 0.3–0.6). However, this is likely to be hindered by the limited power of colocalization. Our analysis showed that it was more likely for the candidate causal proteins and cognitive traits to share a single causal variant than have different causal variants (PP.H4>PP.H3). Fourth, we observed opposite effects of the two ligands of CSF-1 receptor (CSF-1 and IL-34) on general cognitive function, but we were not able to disentangle which interventions would be most effective in the clinical setting. Animal experiments and intervention trials should be considered to bring light to this. In addition, further research is warranted to decipher the role of protein-protein interaction networks in cognitive function. Fifth, this study was based on individuals of European ancestry, thus our findings may not be generalized to other ethnic groups.

## Conclusion

By applying MR analysis and colocalization with a rigorous selection of genetic instrument accounting for both pQTL and brain-specific eQTL, our study investigated the relationship of a wide range of plasma proteins that were known to be related to cardiovascular risk with cognitive traits and found supporting evidence for MPO, CSF-1, CTSD, and CD40. These proteins are involved in shared pathways between CVD and those for cognitive reserve or affecting cognitive decline, suggesting they might be used as therapeutic targets for cognitive impairment. Functional work and further investigations in more relevant tissues are needed.

## Data Availability

Publicly available datasets were analyzed in this study. This data can be found here: https://www.ccace.ed.ac.uk/node/335; https://www.nature.com/articles/s41562-020-00936-2; https://zenodo.org/record/2615265; https://www.decode.com/summarydata/; https://www.gtexportal.org/home/datasets.

## Abbreviation

CVD, Cardiovascular disease; CTSD, Cathepsin D; eQTL, Expression quantitative trait loci; *g* Factor, General factor of intelligence; GWAS, Genome-wide association studies; Genomic SEM, Genomic structural equation modelling; IVW, Inverse-variance weighted; CSF-1, Macrophage colony-stimulating factor 1; MR, Mendelian randomisation; MAF, Minor allele frequency; MPO, Myeloperoxidase; NPX, Normalized protein expression; PP, Posterior probability; pQTL, Protein quantitative trait loci; PEA, Proximity extension assay; SNPs, Single-nucleotide polymorphisms; TNFRSF5, commonly known as CD40, Tumour necrosis factor receptor superfamily member 5; WM, Weighted median.
