# Supplementary material for: Circulatory proteins relate cardiovascular disease to cognitive performance: A mendelian randomisation study
Source: Front Genet. 2023 Feb 17;14:1124431. doi: 10.3389/fgene.2023.1124431 (PMC9981660; doi:10.3389/fgene.2023.1124431)
Supplement: Supplementary file 1 [file DataSheet2.DOCX]

**Circulatory proteins relate cardiovascular disease to cognitive performance: a Mendelian randomisation study**

**Supplementary Figure**

**
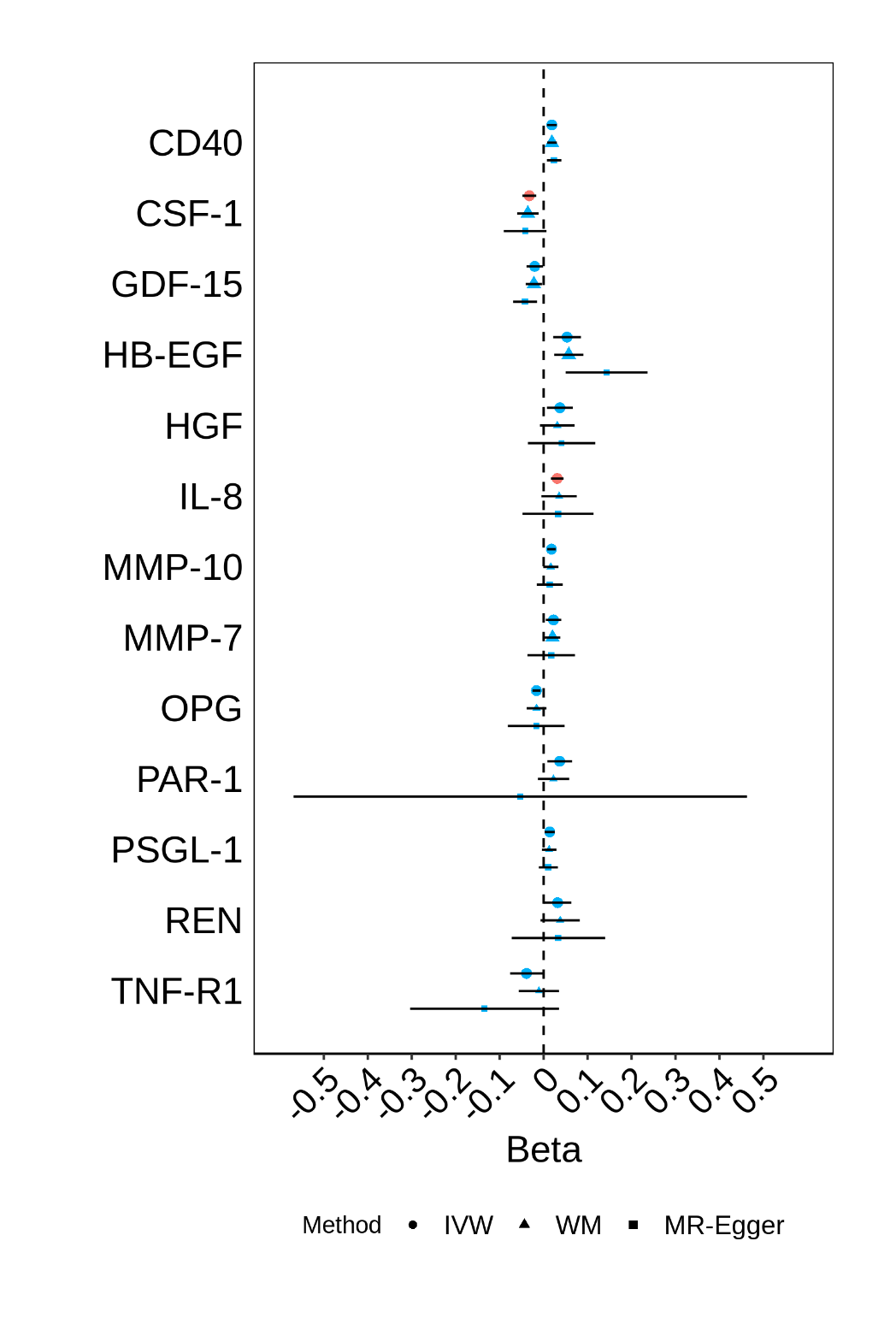
**

Figure S1 Forest plot for the association of pQTL predicted circulatory protein level with general cognitive function (selected findings with P-value<0.05 using IVW). The symbol in red indicates an association with P-value<0.05/180. The symbol in blue and larger in size indicates an association with P-value<0.05. (Inverse variance weighted (IVW); weighted median (WM))


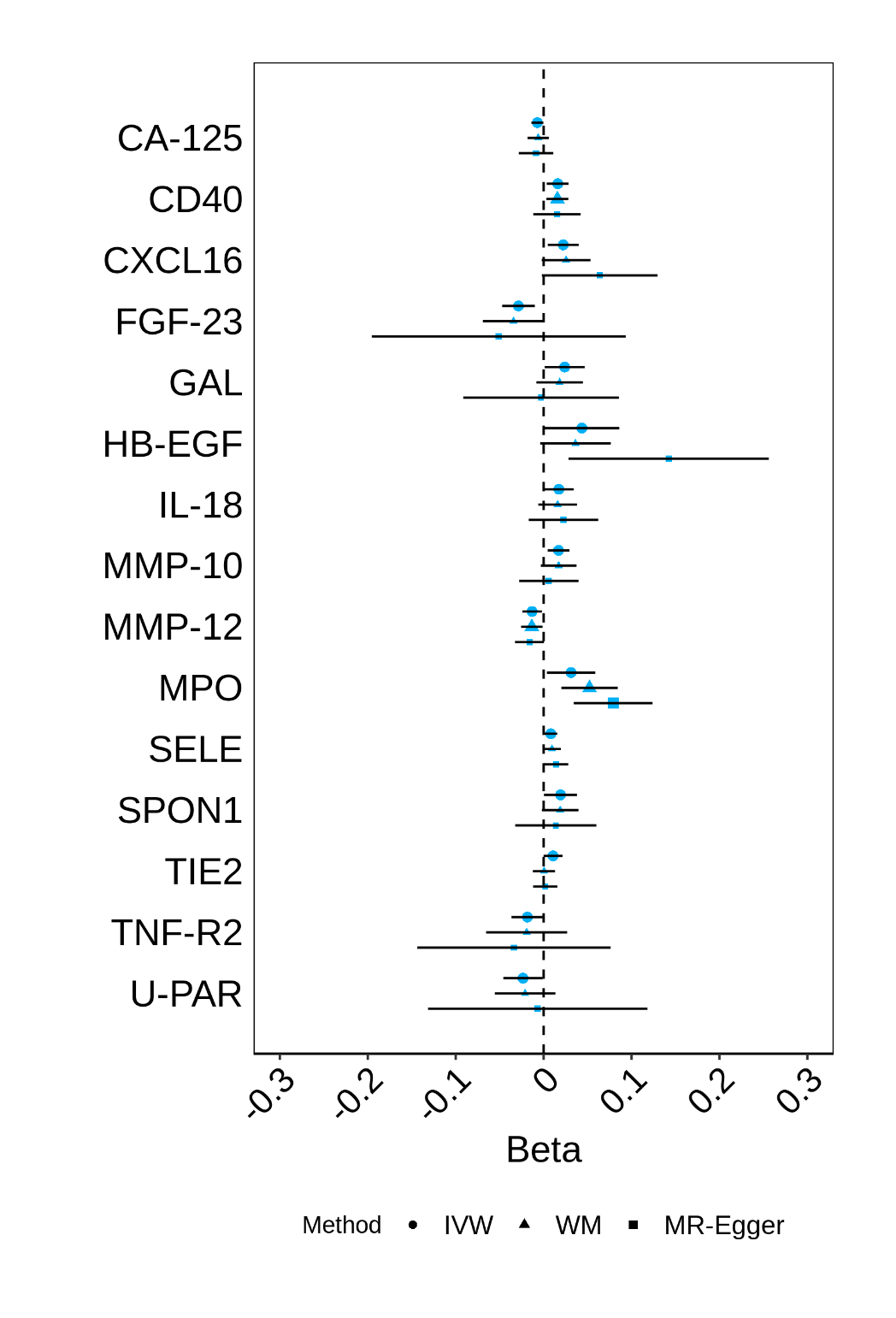


Figure S2 Forest plot for the association of pQTL predicted circulatory protein level with g Factor (selected findings with P-value<0.05 using IVW). The symbol in red indicates an association with P-value<0.05/180. The symbol in blue and larger in size indicates an association with P-value<0.05. (Inverse variance weighted (IVW); weighted median (WM))
